# Correction: MicroRNAs expressed during normal wound healing and their associated pathways: A systematic review and bioinformatics analysis

**DOI:** 10.1371/journal.pone.0294488

**Published:** 2023-11-09

**Authors:** Morgana Lüdtke Azevedo, Roberta Giorgi Silveira, Fernanda Nedel, Rafael Guerra Lund

There are errors in the Funding section. The correct Funding statement is: Financial support for this research was provided by the Brazilian Government Funding Agencies: CAPES–code 001, CAPES/PrInt Program (grnt#88887.310585/2018-0), and CNPq (INOVA GRAFENO-MCTI grant #409092/2022-3). The funders had no role in study design, data collection and analysis, decision to publish, or preparation of the manuscript.
