# Supplementary material for: MicroRNAs expressed during normal wound healing and their associated pathways: A systematic review and bioinformatics analysis
Source: PLoS One. 2023 Apr 13;18(4):e0281913. doi: 10.1371/journal.pone.0281913 (PMC10101427; doi:10.1371/journal.pone.0281913)
Supplement: S1 Table — (DOCX) [file pone.0281913.s002.docx]

**S1 Table. Correlation of results regarding the expression pattern of miRNAs at different times of analysis.**

| **Author, Year** | **Collection time** | **Technique** | **Increased microRNA expression.** | | **Decreased microRNA expression** | | **Not affected** |
| --- | --- | --- | --- | --- | --- | --- | --- |
| Aunin et al., 2017 | Day 3^#^ | RT-qPCR | miR-31 | | miR-200ª | miR-141 | - |
|  |  |  |  |  | miR-200b | miR-429 |  |
|  |  |  |  |  | miR-200c |  |  |
|  |  | Microarray | mmu-miR-705 | mmu-miR-669f-3p | mmu-miR-541-5p | mmu-miR-500-3p | mmu-miR146b-5p |
|  |  |  | mmu-miR-346-3p | mmu-miR-466i-3p | mmu-miR-1894-3p | mmu-miR-467d-3p | mmu-miR-214-3p |
|  |  |  | mmu-miR-1892 | mmu-miR-466m-3p | mmu-miR-5119 | mmu-miR-677-3p | mmu-miR-25-3p |
|  |  |  | mmu-miR-31-5p | mmu-miR-669e-3p | mmu-miR-434-3p | mmu-miR-199a-5p |  |
|  |  |  |  | | mmu-miR-127-3p | mmu-miR-222-3p |  |
|  |  |  |  |  | mmu-miR-200c-3p | mmu-miR-5121 |  |
|  |  |  |  |  | mmu-miR-376b-3p | mmu-miR-22-3p |  |
|  |  |  |  |  | mmu-miR-3970 | mmu-miR-5100 |  |
|  |  |  |  |  | mmu-miR-411-5p | mmu-miR-26b-5p |  |
|  |  |  |  |  | mmu-miR-29b-3p | mmu-miR-3963 |  |
|  |  |  |  |  | mmu-miR-379-5p | mmu-miR-221-3p |  |
|  |  |  |  |  | mmu-miR-329-3p | mmu-miR-25-3p |  |
|  |  |  |  |  | mmu-miR-335-5p | mmu-miR-150-5p |  |
|  | Day 5^#^ | RT-qPCR | miR-31 | | miR-200a | miR-141 | - |
|  |  |  |  |  | miR-200b | miR-429 |  |
|  |  |  |  |  | miR-200c |  |  |
|  |  | Microarray | mmu-miR-5119 | mmu-miR-335-5p | mmu-miR-541-5p | mmu-miR-500-3p | mmu-miR-705 |
|  |  |  | mmu-miR-376b-3p | mmu-miR-677-3p | mmu-miR-1894-3p | mmu-miR-199a-5p | mmu-miR-669f-3p |
|  |  |  | mmu-miR-1892 | mmu-miR-214-3p | mmu-miR-346-3p | mmu-miR-222-3p | mmu-miR-467d-3p |
|  |  |  | mmu-miR-31-5p |  | mmu-miR-434-3p | mmu-miR-466m-3p | mmu-miR-466i-3p |
|  |  |  |  | | mmu-miR-127-3p | mmu-miR-669e-3p | mmu-miR-5100 |
|  |  |  |  |  | mmu-miR-200c-3p | mmu-miR-5121 |  |
|  |  |  |  |  | mmu-miR-3970 | mmu-miR-22-3p |  |
|  |  |  |  |  | mmu-miR-411-5p | mmu-miR-26b-5p |  |
|  |  |  |  |  | mmu-miR-29b-3p | mmu-miR-3963 |  |
|  |  |  |  |  | mmu-miR-379-5p | mmu-miR-221-3p |  |
|  |  |  |  |  | mmu-miR146b-5p | mmu-miR-25-3p |  |
|  |  |  |  |  | mmu-miR-329-3p | mmu-miR-150-5p |  |
|  | Day 7^#^ | RT-qPCR | miR-200a | miR-429 | miR-31 | miR-200c | - |
|  |  |  |  | | miR-200b | miR-141 |  |
|  | Day 3^✢^ | RT-qPCR | miR-200c |  | miR-31 | miR-141 | - |
|  |  |  |  | | miR-200a | miR-429 |  |
|  |  | Microarray | mmu-miR-541-5p | mmu-miR-29b-3p | mmu-miR-411-5p | mmu-miR-466m-3p | mmu-miR-3970 |
|  |  |  | mmu-miR-705 | mmu-miR146b-5p | mmu-miR-31-5p | mmu-miR-669e-3p | mmu-miR-329-3p |
|  |  |  | mmu-miR-1894-3p | mmu-miR-500-3p | mmu-miR-379-5p | mmu-miR-5121 | mmu-miR-199a-5p |
|  |  |  | mmu-miR-5119 | mmu-miR-677-3p | mmu-miR-335-5p | mmu-miR-22-3p |  |
|  |  |  | mmu-miR-346-3p | mmu-miR-222-3p | mmu-miR-669f-3p | mmu-miR-25-3p |  |
|  |  |  | mmu-miR-434-3p | mmu-miR-214-3p | mmu-miR-467d-3p | mmu-miR-150-5p |  |
|  |  |  | mmu-miR-127-3p | mmu-miR-5100 | mmu-miR-466i-3p |  |  |
|  |  |  | mmu-miR-200c-3p | mmu-miR-26b-5p |  | |  |
|  |  |  | mmu-miR-376b-3p | mmu-miR-3963 |  |  |  |
|  |  |  | mmu-miR-1892 | mmu-miR-221-3p |  |  |  |
|  | Day 5^✢^ | RT-qPCR | - | | miR-31 | miR-141 | - |
|  |  |  |  |  | miR-200a | miR-429 |  |
|  |  |  |  |  | miR-200b |  |  |
|  |  |  |  |  | miR-200c |  |  |
|  |  | Microarray | mmu-miR-541-5p | mmu-miR-669f-3p | mmu-miR-1892 |  | - |
|  |  |  | mmu-miR-705 | mmu-miR-467d-3p | mmu-miR-31-5p |  |  |
|  |  |  | mmu-miR-1894-3p | mmu-miR-677-3p | mmu-miR146b-5p |  |  |
|  |  |  | mmu-miR-5119 | mmu-miR-199a-5p | mmu-miR-335-5p |  |  |
|  |  |  | mmu-miR-346-3p | mmu-miR-466i-3p | mmu-miR-222-3p |  |  |
|  |  |  | mmu-miR-434-3p | mmu-miR-466m-3p | mmu-miR-26b-5p |  |  |
|  |  |  | mmu-miR-127-3p | mmu-miR-214-3p | mmu-miR-3963 |  |  |
|  |  |  | mmu-miR-376b-3p | mmu-miR-669e-3p | mmu-miR-221-3p |  |  |
|  |  |  | mmu-miR-3970 | mmu-miR-5121 | mmu-miR-200c-3p |  |  |
|  |  |  | mmu-miR-411-5p | mmu-miR-22-3p |  |  |  |
|  |  |  | mmu-miR-29b-3p | mmu-miR-5100 |  |  |  |
|  |  |  | mmu-miR-379-5p | mmu-miR-25-3p |  |  |  |
|  |  |  | mmu-miR-329-3p | mmu-miR-150-5p |  |  |  |
|  |  |  | mmu-miR-500-3p |  |  |  |  |
|  | Day 7^✢^ | RT-qPCR | miR-31 |  | miR-200a | miR-141 | - |
|  |  |  |  | | miR-200b | miR-429 |  |
|  |  |  |  |  | miR-200c |  |  |
| Chan et al., 2012a | Day 3 | RT-qPCR | - | | miR-199a-5p |  | - |
| Chan et al., 2012b | Day 3 | RT-qPCR | - | | miR-200b |  | - |
|  | Day 7 | RT-qPCR | - | | miR-200b |  | - |
|  | Day 14 | RT-qPCR | - | | miR-200b |  | - |
| Chen et al., 2019 | 6 h | RT-qPCR | mmu-miR-10b-5p | mmu-miR-451a | mmu-miR-21-5p | mmu-miR-126a-5p | - |
|  |  |  | mmu-miR-223-3p | mmu-miR-34c-5p | mmu-miR-31-5p | mmu-miR-199a-5p |  |
|  |  |  | mmu-miR-140-3p | mmu-miR-126a-3p | mmu-miR-24-2-5p |  |  |
|  |  |  | mmu-miR-99a-5p | mmu-miR-125b-5p |  | |  |
|  |  |  | mmu-miR-26a-5p | mmu-miR-125a-5p |  |  |  |
|  | Day 1 | RT-qPCR | mmu-miR-223-3p | mmu-miR-451a | mmu-miR-10b-5p | mmu-miR-126a-5p | - |
|  |  |  | mmu-miR-21-5p | mmu-miR-34c-5p | mmu-miR-99a-5p | mmu-miR-126a-3p |  |
|  |  |  | mmu-miR-140-3p |  | mmu-miR-26a-5p | mmu-miR199a-5p |  |
|  |  |  |  | | mmu-miR-31-5p | mmu-miR-125b-5p |  |
|  |  |  |  |  | mmu-miR-24-2-5p | mmu-miR-125a-5p |  |
|  | Day 5 | RT-qPCR | mmu-miR-233-3p | mmu-miR-451a | mmu-miR-10b-5p | mmu-miR-126a-3p | - |
|  |  |  | mmu-miR-21-5p | mmu-miR-34c-5p | mmu-miR26a-5p | mmu-miR-199b-3p |  |
|  |  |  | mmu-miR-140-3p | mmu-miR-199a-5p | mmu-miR-24-2-5p | mmu-miR-125b-5p |  |
|  |  |  | mmu-miR-99a-5p | mmu-miR-125a-5p | mmu-miR-126a-5p |  |  |
|  |  |  | mmu-miR-31-5p |  |  |  |  |
| Etich et al., 2017 | Day 1 | RT-qPCR | - |  | miR-204 | miR-205 | - |
|  | Day 5 | RT-qPCR | miR-31 |  | miR-204 | miR-205 | - |
|  | Day 7 | RT-qPCR | miR-31 |  | - |  | - |
|  | Day 10 | RT-qPCR | miR-31 |  | - |  | - |
|  | Day 14 | RT-qPCR | miR-31 |  | - |  | - |
| Jin and Chung, 2018 | Day 1 | Microarray | mmu-miR-185 | mmu-miR-139-5p | mmu-miR-152 | mmu-miR-199a-5p | - |
|  |  |  | mmu-miR-151-5p | mmu-miR-1949 | mmu-miR-365 | mmu-miR-127 |  |
|  |  |  | mmu-miR-423-5p | mmu-miR-486 | mmu-miR-125a-5p | mmu-miR-183 |  |
|  |  |  | mmu-miR-92a | mmu-miR-2861 | mmu-let-7d* | mmu-miR-221 |  |
|  |  |  | mmu-miR-92b | mmu-miR-2134 | mmu-miR-181d | mmu-miR-151-3p |  |
|  |  |  | mmu-miR-714 | mmu-miR-2135 | mmu-miR-125b-5p | mmu-miR-689 |  |
|  |  |  | mmu-miR-2137 | mmu-miR-2133 | mmu-miR-30c | mmu-miR-19b |  |
|  |  |  | mmu-miR-361 | mmu-miR-705 | mmu-miR-99a | mmu-miR-200b |  |
|  |  |  | mmu-miR-2141 | mmu-miR-25 | mmu-miR-100 | mmu-miR-200c |  |
|  |  |  | mmu-miR-146b | mmu-miR-1894-3p | mmu-miR-182 | mmu-miR-22 |  |
|  |  |  | mmu-miR-2146 | mmu-miR-652 | mmu-miR-30d | mmu-miR-429 |  |
|  |  |  | mmu-miR-2145 | mmu-miR-15b | mmu-miR-146a | mmu-miR-320 |  |
|  |  |  | mmu-miR-2138 | mmu-miR-223 | mmu-miR-1839-5p | mmu-miR-744 |  |
|  |  |  | mmu-miR-132 | mmu-miR-2138 | mmu-miR-199b* | mmu-miR-690 |  |
|  |  |  | mmu-miR-762 |  | mmu-miR-128 | mmu-miR-10b |  |
|  |  |  |  | | mmu-miR-199a-3p | mmu-miR-98 |  |
|  |  |  |  |  | mmu-miR-1939 | mmu-miR-10a |  |
|  |  |  |  |  | mmu-miR-214 |  |  |
|  |  | RT-qPCR | - | | miR-99a | miR-100 | - |
|  |  |  |  |  | miR-99b |  |  |
|  | Day 5 | Microarray | mmu-miR-152 | mmu-miR-221 | mmu-miR-365 | mmu-miR-128 |  |
|  |  |  | mmu-miR-714 | mmu-miR-1949 | mmu-miR-125a-5p | mmu-miR-132 |  |
|  |  |  | mmu-miR-2137 | mmu-miR-2861 | mmu-miR-99a | mmu-miR-183 |  |
|  |  |  | mmu-miR-199b* | mmu-miR-2134 | mmu-let7d* | mmu-miR-139-5p |  |
|  |  |  | mmu-miR-2141 | mmu-miR-689 | mmu-miR-100 | mmu-miR-151-3p |  |
|  |  |  | mmu-miR-199a-3p | mmu-miR-19b | mmu-miR-181d | mmu-miR-486 |  |
|  |  |  | mmu-miR-1939 | mmu-miR-22 | mmu-miR-30c | mmu-miR-200b |  |
|  |  |  | mmu-miR-2146 | mmu-miR-2135 | mmu-miR-182 | mmu-miR-200c |  |
|  |  |  | mmu-miR-214 | mmu-miR-223 | mmu-miR-185 | mmu-miR-429 |  |
|  |  |  | mmu-miR-2145 | mmu-miR-2133 | mmu-miR-30d | mmu-miR-320 |  |
|  |  |  | mmu-miR-199a-5p | mmu-miR-705 | mmu-miR-151-5p | mmu-miR-744 |  |
|  |  |  | mmu-miR-2138 | mmu-miR-690 | mmu-miR-423-5p | mmu-miR-10b |  |
|  |  |  | mmu-miR-127 | mmu-miR-1894-3p | mmu-miR-92a | mmu-miR-25 |  |
|  |  |  | mmu-miR-762 |  | mmu-miR-146a | mmu-miR-98 |  |
|  |  |  |  | | mmu-miR-365 | mmu-miR-125b-5p |  |
|  |  |  |  |  | mmu-miR-92b | mmu-miR-10a |  |
|  |  |  |  |  | mmu-miR-361 | mmu-miR-652 |  |
|  |  |  |  |  | mmu-miR-1839-5p | mmu-miR-15b |  |
|  |  |  |  |  | mmu-miR-146b |  |  |
|  |  | RT-qPCR | - | | - | | miR-99a |
|  |  |  |  |  |  |  | miR-99b |
|  |  |  |  |  |  |  | miR-100 |
| Shi et al., 2018 | Day 1 | RT-qPCR | miR-31 |  | - |  | - |
|  | Day 2 | RT-qPCR | miR-31 |  | - |  | - |
|  | Day 3 | RT-qPCR | miR-31 |  | - |  | - |
|  | Day 6 | RT-qPCR | miR-31 |  | - |  | - |
|  | Day 8 | RT-qPCR | miR-31 |  | - |  | - |
|  | Day 10 | RT-qPCR | miR-31 |  | - |  | - |
|  | Day 12 | RT-qPCR | - |  | miR-31 |  | - |
|  | Day 17 | RT-qPCR | - |  | miR-31 |  | - |
| Simões et al., 2019 | 6 h | Microarray | mmu-miR-378b | mmu-miR-99a-5p | mmu-miR-362-5p | mmu-miR-30c-2-3p | - |
|  |  |  | mmu-miR-30a-5p | mmu-miR-532-5p | rno-miR-140-3p | mmu-miR-193b-5p |  |
|  |  |  | mmu-miR-21a-5p | mmu-miR-369-5p | mmu-miR-363-3p | cgr-miR-214-5p |  |
|  |  |  | mmu-miR-132-3p | mmu-mir-3470b-p5 | mmu-miR-130b-5p | mmu-miR-10a-5p |  |
|  |  |  | mmu-miR-142a-5p | mmu-miR-133a-3p | mmu-miR-193b-3p | mmu-miR-10a-3p |  |
|  |  |  | mmu-miR-574-5p | hsa-miR-4448 | mmu-miR-3068-5p | mmu-miR-674-5p |  |
|  |  |  | mmu-miR-148b-3p | mmu-miR-708-3p | mmu-miR-31-5p | chi-miR-99b-3p |  |
|  |  |  | mmu-miR-370-3p | mmu-miR-125b-1-3p | mmu-miR-667-3p | mmu-miR-107-3p |  |
|  |  |  | mmu-miR-503-5p | mmu-miR-5099 | mmu-miR-425-3p | mmu-miR-574-3p |  |
|  |  |  | mmu-miR-223-5p | mmu-miR-615-3p | mmu-miR-361-3p | mmu-miR-24-2-5p |  |
|  |  |  | mmu-miR-133a-5p | mmu-miR-340-3p | mmu-miR-26a-5p | mmu-miR-210-3p |  |
|  |  |  | mmu-miR-409-3p | mmu-miR-212-3p | mmu-miR-15b-3p | mmu-miR-484 |  |
|  |  |  | mmu-miR-30e-3p | mmu-miR-192-5p | mmu-miR-152-5p | mmu-miR-15b-5p |  |
|  |  |  | mmu-miR-540-3p | mmu-miR-337-5p | mmu-miR-130b-3p | rno-miR-25-5p |  |
|  |  |  | mmu-miR-106b-3p | mmu-miR-673-5p | mmu-miR-125b-5p | mmu-miR-211-5p |  |
|  |  |  | cgr-miR-139-5p | mmu-miR-146b-3p | mmu-miR-181c-5p | mmu-miR-1247-5p |  |
|  |  |  | mmu-miR-1198-5p | mmu-miR-299b-3p | mmu-miR-669c-5p | mmu-miR-99a-3p |  |
|  |  |  | mmu-miR-7a-5p | mmu-miR-329-5p | mmu-miR-365-3p | mmu-miR-23b-3p |  |
|  |  |  | mmu-miR-1983 | mmu-miR-1195 | ptr-miR-203 | mmu-miR-676-5p |  |
|  |  |  | mmu-miR-195a-5p | mmu-miR-329-3p | mmu-miR-30c-5p | mmu-miR-712-5p |  |
|  |  |  | mmu-miR-485-3p | mmu-miR-3068-3p | mmu-miR-203-3p | hsa-miR-7977 |  |
|  |  |  | mmu-miR-223-3p | mmu-miR-340-5p | mmu-miR-872-3p | mmu-miR-342-3p |  |
|  |  |  | mmu-miR-335-3p | mmu-miR-328-3p | mmu-miR-10b-5p | mmu-miR-542-5p |  |
|  |  |  | mmu-miR-433-3p | mmu-miR-335-5p | rno-miR-214-3p | chi-miR-326-5p |  |
|  |  |  | mmu-miR-30a-3p | mmu-miR-132-5p | mdo-miR-26-5pC | mmu-miR-145a-5p |  |
|  |  |  | mmu-miR-34c-5p | mmu-miR-152-3p | mmu-miR-423-3p | mmu-miR-26a-2-3p |  |
|  |  |  | mmu-miR-298-5p | mmu-miR-434-5p | mdo-miR-26-5p | mmu-miR-425-5p |  |
|  |  |  | mmu-miR-379-3p | mmu-miR-351-5p | mmu-miR-199a-5p | oan-miR-1386 |  |
|  |  |  | mmu-let-7g-5p | mmu-miR-379-5p | mmu-miR-200c-3p | mmu-miR-126a-3p |  |
|  |  |  | mmu-miR-3535 | hsa-miR-4454 | mmu-miR-3473d | mmu-miR-652-3p |  |
|  |  |  | mmu-miR-96-5p | mmu-miR-664-3p | mmu-miR-125a-5p | sha-miR-24 |  |
|  |  |  | hsa-miR-411-3p | mmu-miR-665-3p | mmu-miR-181d-5p | mmu-miR-182-5p |  |
|  |  |  | mmu-miR-134-5p | mmu-miR-148b-5p | mmu-miR-98-3p | mmu-miR-1948-3p |  |
|  |  |  | mmu-miR-503-3p | mmu-miR-20a-5p | mmu-miR-221-3p | mmu-miR-8112 |  |
|  |  |  | mmu-miR-351-3p | rno-miR-181a-2-3p |  | |  |
|  |  |  | mmu-miR-30d-5p | mmu-miR-221-5p |  |  |  |
|  |  |  | mmu-miR-467b-5p | rno-miR-378a-5p |  |  |  |
|  |  |  | mmu-miR-1a-3p | mmu-mir-3102-p3 |  |  |  |
|  |  |  | mmu-miR-323-3p | rno-miR-874-3p |  |  |  |
|  |  |  | mmu-miR-541-5p | mmu-miR-1839-5p |  |  |  |
|  |  |  | mmu-miR-494-3p | mmu-miR-3970 |  |  |  |
|  |  |  | mmu-miR-376b-3p | mmu-miR-495-3p |  |  |  |
|  |  |  | mmu-miR-186-5p | mmu-miR-300-3p |  |  |  |
|  |  |  | mmu-miR-212-5p | mmu-miR-28c |  |  |  |
|  |  |  | rno-miR-25-3p | mmu-miR-224-5p |  |  |  |
|  |  |  | mmu-miR-543-3p | mmu-miR-382-3p |  |  |  |
|  |  |  | mmu-miR-204-5p | mmu-miR-222-3p |  |  |  |
|  |  |  | mmu-miR-200a-3p | mmu-miR-582-3p |  |  |  |
|  |  |  | cfa-miR-194 | mmu-miR-496a-3p |  |  |  |
|  |  |  | mmu-miR-34b-3p | mmu-miR-409-5p |  |  |  |
|  |  |  | mmu-miR-450b-5p | mmu-miR-194-5p |  |  |  |
|  |  |  | mmu-mir-3470a-p3 | mmu-miR-6538 |  |  |  |
|  |  |  | mmu-miR-679-5p | bta-miR-378 |  |  |  |
|  |  |  | cgr-miR-139-3p | mmu-miR-17-5p |  |  |  |
|  |  |  | mmu-miR-125a-3p | mmu-miR-144-3p |  |  |  |
|  |  |  | hsa-mir-4450-p3 | mmu-miR-5126 |  |  |  |
|  |  |  | mmu-miR-382-5p | mmu-miR-450a-5p |  |  |  |
|  |  |  | mmu-miR-877-5p | mmu-miR-467c-5p |  |  |  |
|  |  |  | mmu-miR-145a-3p | mmu-miR-378d |  |  |  |
|  |  |  | mmu-miR-1193-3p | mmu-miR-143-3p |  |  |  |
|  |  |  | mmu-miR-21a-3p | mmu-miR-23a-5p |  |  |  |
|  |  |  | hsa-miR-320b | mmu-miR-410-3p |  |  |  |
|  |  |  | mmu-miR-361-5p | mmu-miR-191-3p |  |  |  |
|  |  |  | mmu-miR-1a-1-5p | mmu-miR-222-5p |  |  |  |
|  |  |  | mdo-miR-22-3p | hsa-miR-1261 |  |  |  |
|  |  |  | ptr-let-7i | mmu-miR-434-3p |  |  |  |
|  | Day 1 | Microarray | mmu-miR-362-5p | mmu-miR-125b-1-3p | mmu-miR-30a-5p | mmu-miR-329-5p |  |
|  |  |  | mmu-miR-378b | mmu-miR-5099 | mmu-miR-193b-3p | mdo-miR-26-5p |  |
|  |  |  | mmu-miR-21a-5p | mmu-miR-340-3p | mmu-miR-133a-5p | mmu-miR-199a-5p |  |
|  |  |  | rno-miR-140-3p | mmu-miR-212-3p | mmu-miR-30e-3p | mmu-miR-329-3p |  |
|  |  |  | mmu-miR-132-3p | mmu-miR-192-5p | mmu-miR-31-5p | mmu-miR-3068-3p |  |
|  |  |  | mmu-miR-142a-5p | mmu-miR-337-5p | mmu-miR-195a-5p | mmu-miR-125a-5p |  |
|  |  |  | mmu-miR-574-5p | mmu-miR-146b-3p | mmu-miR-667-3p | mmu-miR-181d-5p |  |
|  |  |  | mmu-miR-363-3p | mmu-miR-299b-3p | mmu-miR-335-3p | mmu-miR-98-3p |  |
|  |  |  | mmu-miR-148b-3p | mmu-miR-423-3p | mmu-miR-30a-3p | mmu-miR-335-5p |  |
|  |  |  | mmu-miR-370-3p | mmu-miR-1195 | mmu-miR-379-3p | mmu-miR-30c-2-3p |  |
|  |  |  | mmu-miR-503-5p | mmu-miR-200c-3p | mmu-miR-3535 | mmu-miR-193b-5p |  |
|  |  |  | mmu-miR-130b-5p | mmu-miR-3473d | mmu-miR-96-5p | cgr-miR-214-5p |  |
|  |  |  | mmu-miR-223-5p | mmu-miR-340-5p | hsa-miR-411-3p | mmu-miR-10a-5p |  |
|  |  |  | mmu-miR-409-3p | mmu-miR-328-3p | mmu-miR-30d-5p | mmu-miR-434-5p |  |
|  |  |  | mmu-miR-540-3p | mmu-miR-221-3p | mmu-miR-1a-3p | mmu-miR-351-5p |  |
|  |  |  | mmu-miR-106b-3p | mmu-miR-132-5p | mmu-miR-26a-5p | mmu-miR-664-3p |  |
|  |  |  | cgr-miR-139-5p | mmu-miR-152-3p | mmu-miR-494-3p | mmu-miR-107-3p |  |
|  |  |  | mmu-miR-3068-5p | mmu-miR-10a-3p | mmu-miR-376b-3p | mmu-miR-574-3p |  |
|  |  |  | mmu-miR-1198-5p | mmu-miR-674-5p | mmu-miR-186-5p | mmu-miR-24-2-5p |  |
|  |  |  | mmu-miR-7a-5p | chi-miR-99b-3p | mmu-miR-204-5p | mmu-miR-484 |  |
|  |  |  | mmu-miR-1983 | mmu-miR-379-5p | mmu-miR-679-5p | mmu-miR-221-5p |  |
|  |  |  | mmu-miR-485-3p | hsa-miR-4454 | mmu-miR-152-5p | rno-miR-378a-5p |  |
|  |  |  | mmu-miR-223-3p | mmu-miR-665-3p | mmu-miR-125b-5p | rno-miR-874-3p |  |
|  |  |  | mmu-miR-425-3p | mmu-miR-148b-5p | mmu-miR-145a-3p | mmu-miR-211-5p |  |
|  |  |  | mmu-miR-433-3p | mmu-miR-20a-5p | mmu-miR-1193-3p | mmu-miR-495-3p |  |
|  |  |  | mmu-miR-34c-5p | rno-miR-181a-2-3p | mmu-miR-181c-5p | mmu-miR-99a-3p |  |
|  |  |  | mmu-miR-298-5p | mmu-miR-210-3p | mmu-miR-365-3p | mmu-miR-23b-3p |  |
|  |  |  | mmu-let-7g-5p | mmu-miR-15b-5p | mmu-miR-1a-1-5p | mmu-miR-676-5p |  |
|  |  |  | mmu-miR-134-5p | mmu-mir-3102-p3 | ptr-miR-203 | mmu-miR-342-3p |  |
|  |  |  | mmu-miR-503-3p | rno-miR-25-5p | mmu-miR-30c-5p | mmu-miR-409-5p |  |
|  |  |  | mmu-miR-351-3p | mmu-miR-1839-5p | mmu-miR-99a-5p | mmu-miR-145a-5p |  |
|  |  |  | mmu-miR-467b-5p | mmu-miR-3970 | mmu-miR-203-3p | mmu-miR-26a-2-3p |  |
|  |  |  | mmu-miR-323-3p | mmu-miR-1247-5p | mmu-miR-133a-3p | mmu-miR-126a-3p |  |
|  |  |  | mmu-miR-361-3p | mmu-miR-300-3p | mmu-miR-872-3p | mmu-miR-143-3p |  |
|  |  |  | mmu-miR-541-5p | mmu-miR-28c | mmu-miR-10b-5p | mmu-miR-410-3p |  |
|  |  |  | mmu-miR-212-5p | mmu-miR-224-5p | mmu-miR-708-3p | sha-miR-24 |  |
|  |  |  | mmu-miR-15b-3p | mmu-miR-712-5p | rno-miR-214-3p | mmu-miR-182-5p |  |
|  |  |  | rno-miR-25-3p | mmu-miR-382-3p | mdo-miR-26-5pC | mmu-miR-1948-3p |  |
|  |  |  | mmu-miR-543-3p | mmu-miR-222-3p | mmu-miR-615-3p | mmu-miR-8112 |  |
|  |  |  | mmu-miR-200a-3p | hsa-miR-7977 | mmu-miR-673-5p | mmu-miR-434-3p |  |
|  |  |  | cfa-miR-194 | mmu-miR-582-3p |  | |  |
|  |  |  | mmu-miR-34b-3p | mmu-miR-496a-3p |  |  |  |
|  |  |  | mmu-miR-450b-5p | mmu-miR-194-5p |  |  |  |
|  |  |  | mmu-mir-3470a-3p | mmu-miR-6538 |  |  |  |
|  |  |  | cgr-miR-139-3p | bta-miR-378 |  |  |  |
|  |  |  | mmu-miR-125a-3p | mmu-miR-17-5p |  |  |  |
|  |  |  | hsa-mir-4450-p3 | mmu-miR-542-5p |  |  |  |
|  |  |  | mmu-miR-382-5p | mmu-miR-144-3p |  |  |  |
|  |  |  | mmu-miR-877-5p | mmu-miR-5126 |  |  |  |
|  |  |  | mmu-miR-130b-3p | mmu-miR-450a-5p |  |  |  |
|  |  |  | mmu-miR-669c-5p | chi-miR-326-5p |  |  |  |
|  |  |  | mmu-miR-21a-3p | mmu-miR-467c-5p |  |  |  |
|  |  |  | hsa-miR-320b | mmu-miR-378d |  |  |  |
|  |  |  | mmu-miR-361-5p | mmu-miR-425-5p |  |  |  |
|  |  |  | mdo-miR-22-3p | oan-miR-1386 |  |  |  |
|  |  |  | ptr-let-7i | mmu-miR-23a-5p |  |  |  |
|  |  |  | mmu-miR-532-5p | mmu-miR-652-3p |  |  |  |
|  |  |  | mmu-miR-369-5p | mmu-miR-191-3p |  |  |  |
|  |  |  | mmu-mir-3470b-p5 | mmu-miR-222-5p |  |  |  |
|  |  |  | hsa-miR-4448 | hsa-miR-1261 |  |  |  |
|  | Day 5  Microarray | | mmu-miR-362-5p | rno-miR-214-3p | mmu-miR-30a-5p | mdo-miR-26-5p | - |
|  |  |  | mmu-miR-378b | mmu-miR-615-3p | mmu-miR-193b-3p | mmu-miR-3068-3p |  |
|  |  |  | mmu-miR-21a-5p | mmu-miR-340-3p | mmu-miR-133a-5p | mmu-miR-125a-5p |  |
|  |  |  | rno-miR-140-3p | mmu-miR-212-3p | mmu-miR-30e-3p | mmu-miR-328-3p |  |
|  |  |  | mmu-miR-132-3p | mmu-miR-337-5p | mmu-miR-195a-5p | mmu-miR-181d-5p |  |
|  |  |  | mmu-miR-142a-5p | mmu-miR-673-5p | mmu-miR-30a-3p | mmu-miR-30c-2-3p |  |
|  |  |  | mmu-miR-574-5p | mmu-miR-146b-3p | mmu-miR-3535 | mmu-miR-193b-5p |  |
|  |  |  | mmu-miR-363-3p | mmu-miR-299b-3p | mmu-miR-96-5p | mmu-miR-10a-5p |  |
|  |  |  | mmu-miR-148b-3p | mmu-miR-329-5p | mmu-miR-30d-5p | mmu-miR-664-3p |  |
|  |  |  | mmu-miR-370-3p | mmu-miR-423-3p | mmu-miR-1a-3p | mmu-miR-107-3p |  |
|  |  |  | mmu-miR-503-5p | mmu-miR-1195 | mmu-miR-26a-5p | mmu-miR-574-3p |  |
|  |  |  | mmu-miR-130b-5p | mmu-miR-199a-5p | mmu-miR-186-5p | rno-miR-181a-2-3p |  |
|  |  |  | mmu-miR-223-5p | mmu-miR-200c-3p | mmu-miR-204-5p | mmu-miR-24-2-5p |  |
|  |  |  | mmu-miR-409-3p | mmu-miR-329-3p | mmu-miR-200a-3p | mmu-miR-484 |  |
|  |  |  | mmu-miR-540-3p | mmu-miR-3473d | mmu-miR-125b-5p | mmu-miR-221-5p |  |
|  |  |  | mmu-miR-106b-3p | mmu-miR-340-5p | mmu-miR-145a-3p | rno-miR-378a-5p |  |
|  |  |  | cgr-miR-139-5p | mmu-miR-98-3p | mmu-miR-181c-5p | mmu-miR-1839-5p |  |
|  |  |  | mmu-miR-3068-5p | mmu-miR-335-5p | hsa-miR-320b | mmu-miR-211-5p |  |
|  |  |  | mmu-miR-1198-5p | mmu-miR-221-3p | mmu-miR-365-3p | mmu-miR-99a-3p |  |
|  |  |  | mmu-miR-7a-5p | mmu-miR-132-5p | mmu-miR-1a-1-5p | mmu-miR-23b-3p |  |
|  |  |  | mmu-miR-1983 | cgr-miR-214-5p | ptr-miR-203 | mmu-miR-676-5p |  |
|  |  |  | mmu-miR-31-5p | mmu-miR-152-3p | mmu-miR-30c-5p | mmu-miR-145a-5p |  |
|  |  |  | mmu-miR-485-3p | mmu-miR-10a-3p | mmu-miR-99a-5p | mmu-miR-26a-2-3p |  |
|  |  |  | mmu-miR-223-3p | mmu-miR-674-5p | mmu-miR-203-3p | mmu-miR-378d |  |
|  |  |  | mmu-miR-667-3p | mmu-miR-434-5p | mmu-mir-3470b-p5 | mmu-miR-143-3p |  |
|  |  |  | mmu-miR-425-3p | chi-miR-99b-3p | mmu-miR-133a-3p | mmu-miR-652-3p |  |
|  |  |  | mmu-miR-335-3p | mmu-miR-351-5p | mmu-miR-10b-5p | sha-miR-24 |  |
|  |  |  | mmu-miR-433-3p | mmu-miR-379-5p | mmu-miR-708-3p | mmu-miR-182-5p |  |
|  |  |  | mmu-miR-34c-5p | hsa-miR-4454 | mdo-miR-26-5pC | mmu-miR-8112 |  |
|  |  |  | mmu-miR-298-5p | mmu-miR-665-3p | mmu-miR-192-5p |  |  |
|  |  |  | mmu-miR-379-3p | mmu-miR-148b-5p |  | |  |
|  |  |  | mmu-let-7g-5p | mmu-miR-20a-5p |  |  |  |
|  |  |  | hsa-miR-411-3p | mmu-miR-210-3p |  |  |  |
|  |  |  | mmu-miR-134-5p | mmu-miR-15b-5p |  |  |  |
|  |  |  | mmu-miR-503-3p | mmu-mir-3102-p3 |  |  |  |
|  |  |  | mmu-miR-351-3p | rno-miR-25-5p |  |  |  |
|  |  |  | mmu-miR-467b-5p | rno-miR-874-3p |  |  |  |
|  |  |  | mmu-miR-323-3p | mmu-miR-3970 |  |  |  |
|  |  |  | mmu-miR-361-3p | mmu-miR-495-3p |  |  |  |
|  |  |  | mmu-miR-541-5p | mmu-miR-1247-5p |  |  |  |
|  |  |  | mmu-miR-494-3p | mmu-miR-300-3p |  |  |  |
|  |  |  | mmu-miR-376b-3p | mmu-miR-28c |  |  |  |
|  |  |  | mmu-miR-212-5p | mmu-miR-224-5p |  |  |  |
|  |  |  | mmu-miR-15b-3p | mmu-miR-712-5p |  |  |  |
|  |  |  | rno-miR-25-3p | mmu-miR-382-3p |  |  |  |
|  |  |  | mmu-miR-543-3p | mmu-miR-222-3p |  |  |  |
|  |  |  | cfa-miR-194 | hsa-miR-7977 |  |  |  |
|  |  |  | mmu-miR-34b-3p | mmu-miR-582-3p |  |  |  |
|  |  |  | mmu-miR-450b-5p | mmu-miR-342-3p |  |  |  |
|  |  |  | mmu-mir-3470a-p3 | mmu-miR-496a-3p |  |  |  |
|  |  |  | mmu-miR-679-5p | mmu-miR-409-5p |  |  |  |
|  |  |  | cgr-miR-139-3p | mmu-miR-194-5p |  |  |  |
|  |  |  | mmu-miR-125a-3p | mmu-miR-6538 |  |  |  |
|  |  |  | hsa-miR-4450-p3 | bta-miR-378 |  |  |  |
|  |  |  | mmu-miR-382-5p | mmu-miR-17-5p |  |  |  |
|  |  |  | mmu-miR-152-5p | mmu-miR-542-5p |  |  |  |
|  |  |  | mmu-miR-877-5p | mmu-miR-144-3p |  |  |  |
|  |  |  | mmu-miR-130b-3p | mmu-miR-5126 |  |  |  |
|  |  |  | mmu-miR-1193-3p | mmu-miR-450a-5p |  |  |  |
|  |  |  | mmu-miR-669c-5p | chi-miR-326-5p |  |  |  |
|  |  |  | mmu-miR-21a-3p | mmu-miR-467c-5p |  |  |  |
|  |  |  | mmu-miR-361-5p | mmu-miR-425-5p |  |  |  |
|  |  |  | mdo-miR-22-3p | oan-miR-1386 |  |  |  |
|  |  |  | ptr-let-7i | mmu-miR-126a-3p |  |  |  |
|  |  |  | mmu-miR-532-5p | mmu-miR-23a-5p |  |  |  |
|  |  |  | mmu-miR-369-5p | mmu-miR-410-3p |  |  |  |
|  |  |  | mmu-miR-872-3p | mmu-miR-222-5p |  |  |  |
|  |  |  | hsa-miR-4448 | mmu-miR-1948-3p |  |  |  |
|  |  |  | mmu-miR-125b-1-3p | hsa-miR-1261 |  |  |  |
|  |  |  | mmu-miR-5099 | mmu-miR-434-3p |  |  |  |
| van Solingen et al., 2014 | Day 10 | RT-qPCR | miR-155 | miR-126 | - | | - |
|  |  |  | miR-33 |  |  |  |  |
| Wang et al., 2012 | Day 7 | Microarray | hsa-miR-31 | hsa-miR-29b | mmu-miR376c | mmu-miR-434-5p | - |
|  |  |  | mmu-miR-712* | hsa-miR-373 | hsa-miR-130a | hsa-miR-422b |  |
|  |  |  | mmu-miR-503 | hsa-miR-203 | hsa-miR-181a | hsa-miR-193b |  |
|  |  |  | mmu-miR-696 | hsa-miR-18b | mmu-miR-434-3p | hsa-miR-30a-3p |  |
|  |  |  | hsa-miR-21 | hsa-let-7f | hsa-miR-182 | hsa-miR-181d |  |
|  |  |  | mmu-miR-712 | hsa-miR19a | hsa-miR-181c | hsa-miR-497 |  |
|  |  |  | hsa-miR-451 | hsa-miR-130b | hsa-miR-127 | mmu-miR-676 |  |
|  |  |  | hsa-miR-296 | hsa-miR-16 | hsa-miR-96 | rno-miR-429 |  |
|  |  |  | mmu-mir-714 | hsa-miR-572 | hsa-miR-200b | *mmu-miR-133a |  |
|  |  |  | hsa-miR-223 | hsa-miR-26b | hsa-miR-204 | rno-miR-329 |  |
|  |  |  | hsa-miR-142-5p | hsa-miR-17-3p | hsa-miR-30a-5p |  |  |
|  |  |  | hsa-miR-205 | hsa-miR-365 |  | |  |
|  |  |  | mmu-miR-702 | hsa-miR-125b |  |  |  |
|  |  |  | hsa-miR-612 |  |  |  |  |
|  |  | RT-qPCR | miR-31 | miR-203 | miR-429 | | - |
|  |  |  | miR-21 |  |  |  |  |
|  |  |  |  |  |  | |  |
| Wang et al., 2019 | Day 1 | RT-qPCR | miR-155 | | - | | miR-196 |
|  |  |  |  |  |  |  | miR-142 |
|  | Day 3 | RT-qPCR | miR-196 | miR-155 | - | | - |
|  |  |  | miR-142 |  |  |  |  |
|  | Day 7 | RT-qPCR | miR-196 | miR-155 | - | | miR-142 |
| Zhao et al., 2020 | Day 3 | RT-qPCR | - | | miR-34a | | - |
|  | Day 7 | RT-qPCR | - | | - | | miR-34a |
|  | Day 14 | RT-qPCR | - | | - | | miR-34a |

Main findings of the studies include collection time, miRNA expression technique, and miRNA expression (increased, decreased, or non-affected expression). The tabulated miRNA nomenclature was transcribed exactly as mentioned in the included studies.

^#^ Data referring to young mice (8-week-old).

^+^ Data referring to adult mice (2-year-old).
