# Supplementary material for: MicroRNAs expressed during normal wound healing and their associated pathways: A systematic review and bioinformatics analysis
Source: PLoS One. 2023 Apr 13;18(4):e0281913. doi: 10.1371/journal.pone.0281913 (PMC10101427; doi:10.1371/journal.pone.0281913)
Supplement: Attachment — Submitted filename: PONE-D-21-30551 review report.pdf [file pone.0281913.s004.pdf]

Manuscript number: PONE-D-21-30551
Manuscript title: MicroRNAs expressed during wound healing and their associated pathways: a systematic review and bioinformatics analysis

The systematic review by Azevedo et al. reported numerous miRNAs to regulate different phases of wound healing, in particular the inflammation and proliferation phases. The outcome of this review maybe used to guide preclinical and clinical studies on the role of miRNA in wound healing. In my view, the manuscript cannot be published in its present form.

Major comments:

1. Numerous typographical and grammatical errors have been observed. The English, formulation of certain sentences, and paragraphs need huge improvement to provide readers better understandability of the text.
2. Improvise abstract to provide gist of the manuscript.
3. Although the authors mentioned to include all articles involving *in vivo* animal studies, but only mice studies were included in table 1. Were there no studies using rats or rabbit?
4. Additionally, some points that need further clarification:
    - In table 1, difference between back skin and dorsal skin?
    - Sources of miRNAs, were the miRNAs extracted from peripheral blood or wound tissues?
5. It would be beneficial if the authors include these in the discussion:
    - Comparison between miRNA signatures between day 1 and day 5.
    - Relate the miRNA signatures or union pathways to the inflammatory and proliferation phases of wound healing.
