# Supplementary material for: MicroRNAs expressed during normal wound healing and their associated pathways: A systematic review and bioinformatics analysis
Source: PLoS One. 2023 Apr 13;18(4):e0281913. doi: 10.1371/journal.pone.0281913 (PMC10101427; doi:10.1371/journal.pone.0281913)
Supplement: Attachment — Submitted filename: Azevedo et al PLOS ONE.docx [file pone.0281913.s003.docx]

**MicroRNAs expressed during wound healing and their associated pathways: a systematic review and bioinformatics analysis**

The authors present a timely and interesting analysis of miRNAs associated with wound healing in mouse skin. As the work is focused on physiological healing in healthy mice, perhaps the title needs to reflect this: **MicroRNAs expressed during normal wound healing...**

The manuscript needs editing for English language usage.

**RESULTS**

It is not entirely clear how the authors went from 2,370 articles with titles and abstracts to 915 articles for full text reading. If this this based on >1,400 articles either not being in English or not freely available, this needs to be made clear.

The authors state that day 3 was analysed by only one of the selected articles (page 10, line 188,189). But there are at least 6 studies in Table 2 that have a day 3 collection time: Aunin et al 2017, Chan et al 2012a, 2012b, Shi et al 2018, Wang et al 2019; Zhao et al 2020. So the authors need to remove or clarify that comment about day 3 analysis.

Looking at the day 1 and day 5 pathways for miRNA with increased expression on day 1 and day 5 (Tables 3 and 5), it might be useful to show the overlap between the pathways (e.g. using a Venn diagram). This would shed light on pathways that feature at both times points and delineate them from those that are limited to day 1 or day 5. Same argument goes for data in Tables 4 and 6, for miRNAs with reduced expression.

There were 273 miRNAs for day 1 and 327 for day 5 (p21, line 196ff). Conceivably all the 273 day 1 miRNAs featured in the 327 at day 5, but it would be good to make this clear or else generate a Venn diagram showing the overlap between the two datasets.

**DISCUSSION**

While the comments link miRNAs and targets to wound healing, there needs to be deeper consideration of the relationships between the miRNAs and the transcripts. Consider ErbB and MAPK signalling (p31,lines 338 ff). What makes these pathways noteworthy is not just that there are 18 and 39 target genes, respectively (Table 6) but rather the fact that they feature in the union pathways for miRNAs with DECREASED expression – that is, the targets themselves are likely to be upregulated to support cell proliferation given that the expression of their miRNA regulators is reduced. The same logic applies to most of the pathways considered.

The above also raises further questions: should additional/supplementary Results tables be generated showing the names of the target genes for the “interesting” pathways – “interesting” pathways being the ones considered in the Discussion?

In a similar vein, as presented, we cannot tell which miRNAs are associated with a given pathway. I am not sure if there is a way that allows this to be done easily but that seems to be the missing link between Table 2 and Tables 3-6. If not for all the targets, such insight might at least be relevant for those targets at the overlap/intersection of the day 1 and day 5 data that I mentioned in the Results.

**Minor**

The Simoes et al data in particular (in Table 2) have multiple entries that are not mouse miRNAs (i.e. not prefixed with mmu). This needs some explanation

The papers in Tables 1 and 2, cited with author names, need the corresponding reference number from the bibliography for ease of cross-referencing.

Tables 3-5 have some pathways in bold. It is not clear what the bold font is supposed to signify, if anything.

Page 21,line 204: Table 2 rather than Table 4?

I could see Figure titles but the manuscript does not seem to have included figure legends.
